# Expression of COX2 and p53 in Rat Esophageal Cancer Induced by Reflux of Duodenal Contents

**DOI:** 10.5402/2012/914824

**Published:** 2012-01-05

**Authors:** Naoki Hashimoto

**Affiliations:** Department of Surgery, Kinki University, 377-2 Ohno-Higashi, Osaka Sayama, Osaka 589-8511, Japan

## Abstract

*Aim*. Reflux of duodenal contents can induce mucosal injury, stimulate cell proliferation, and promote tumorigenesis. We examined the expression of COX2 and p53 in rat esophageal lesions induced by duodenal content reflux. *Methods*. Thirty 8-week-old male Wistar rats were exposed to duodenal content esophageal reflux. All animals underwent an esophagoduodenal anastomosis (EDA) with total gastrectomy in order to produce chronic esophagitis. Ten rats were the sham. *Control*. They were sacrificed at the 40th week. Their esophagi were examined for HE, COX2, p53, and proliferating cell nuclear antigen (PCNA). *Results*. After 40 weeks of reflux, dysplasia, squamous cell carcinoma (SCC), and adenocarcinoma (ADC) were found. PCNA labeling index was higher in dysplastic and cancer tissue than that in normal. Overexpression of COX2 was shown in ADC and SCC. Wild-type p53 accumulation was found in ADC, and not in SCC. *Conclusion*. Reflux of duodenal contents into the esophagus led to ADC and SCC in rats. COX2 may play an important role in esophageal cancer by duodenal content reflux. Our present results suggest an association between wild-type p53 accumulation and COX2 expression in ADC, with no such relation seen in SCC.

## 1. Introduction

Esophageal cancer ranks the 10th most common cancer worldwide. Esophageal squamous cell carcinoma is the predominant histological subtype of esophageal cancer. Many genes have recently been reported to be overexpressed during esophageal cancer development. Tumorigenesis is increasingly recognized as a process that involves the coordinated action of a group of genes, rather than a single gene [1]. These genes and the associated control mechanisms may represent potential targets for the prognosis and therapy of esophageal cancer.

Alteration in the p53 tumor suppressor gene seems to be one of the most important events in human cancer. p53 is known to play a central role in sensing and signaling for growth arrest and apoptosis in cells with DNA damage. There is overwhelming evidence that p53 gene alterations are early and frequent events in esophageal cancer and that this gene is associated with the malignant transformation of Barrett's esophagus [2].

Cyclooxygenase2 (COX2) affects many processes including apoptosis, proliferation, angiogenesis, invasiveness, immunosuppression, and inflammation, which are important in carcinogenesis, and is therefore an attractive therapeutic target. Increased expression of COX2 was found in many premalignant tissues and malignant tumors [3]. COX2 is not only becoming a sensitive marker for high-grade squamous epithelial dysplasia of the esophagus and of Barrett's esophagus but also a target for the therapy and prevention of esophageal cancer.

Although many studies have been performed to assess the possible pivotal role of p53 and COX2 in esophageal cancer progression, there are few on the mutual relationships between p53 and COX2 from the standpoint of a series of mechanisms. Therefore, we conducted an immunohistochemical evaluation of the expression of p53 and COX2 in esophageal cancer induced by chronic duodenal reflux.

## 2. Material and Methods

**Figure 1:**
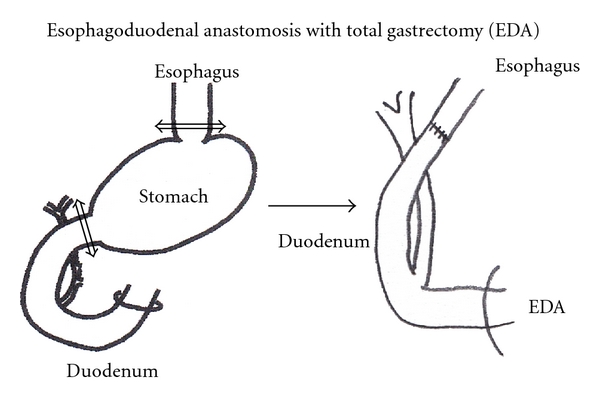
EDA model: Esophagoduodenal anastomosis with total gastrectomy.

Eight-week-old male Wistar rats with a body weight of about 300 g were used for the experiments. They were allowed to acclimate for 2 weeks prior to surgery. Solid food was withdrawn 1 day before and for 1 day after surgery. In 30 rats EDA was performed under general anesthesia (pentobarbital 50 mg/kg body wt ip) through an upper midline incision. The gastroesophageal junction was ligated and the distal esophagus was transected 2 mm above the ligature. Moreover, the gastroduodenal junction was also ligated and the proximal duodenum was transected 3 mm distal to the pylorus. A total gastrectomy was performed with the removal of the entire stomach and end-to-end anastomosis of the esophagus and duodenum. The abdominal incision was closed in two layers (Figure 1). In 10 rats the sham operation induced a midline laparotomy alone (normal control group). Postoperatively the rats were allowed to drink water after six hours and were fed the following day. This procedure was approved by the Animal Care and Facilities Committee, Kinki University.

All the rats were killed as described previously. Special care was taken to separate the esophagus from the duodenum based on the suture line. For the animals killed at the 40th week, all the esophagi were cut longitudinally and were fixed in 10% buffered formalin. The formalin-fixed esophagus was Swiss-rolled, processed, and embedded in paraffin. Five-micron sections were mounted onto glass slides and used for pathological and immunohistochemical analysis.

### 2.1. Immunohistochemistry

**COX2:**

Localization of COX2 protein was determined by immunohistochemical staining using specific antibodies. The EnVision system (Dako, Denmark) was used with autoclave acceleration. Deparaffinized 5 *μ*m sections of a formalin-fixed, paraffin-embedded block were immersed in absolute methanol containing 0.3% hydrogen peroxidase and then covered with normal goat serum (1 : 30). Sections were incubated overnight at 4°C with primary antibody to rat COX2 protein (diluted 1 : 100) (Transduction Laboratory, Lexington, KY). The sections were treated with labeled polymer (Dako) for 2 h. Immersing the sections in 3,3′-diaminobenzil tetrahydrochloride developed the reaction products. The slides were counterstained lightly with hematoxylin.

PCNA (proliferating cell nuclear antigen): Immunohistochemical detection of the PCNA was performed by the avidin-biotin complex method using mouse monoclonal anti-human PCNA antibody and appropriate Histostain Gold AEC kit. The PCNA labeling index has been widely used for the assessment of cell proliferation. In this study, the index was defined as the number of squamous epithelial cells with a PCNA-positive nucleus (or nuclei)/100 squamous epithelial cells (%).

**p53:**

immunohistochemistry staining was performed using the Ventana Benchmark automated immunostainer following the protocols provided by the manufacture. The antibody used was DO-7 for wild-type p53.

### 2.2. Statistical Analysis

Data are expressed as mean ± SD of each group. Analysis of Student's *t*-test was used for statistical analysis. *P* < 0.05 was considered statistically significant.

## 3. Results

### 3.1. General Observation

A total of 37 of 40 (92.5%) rats completed the study. In the EDA group, 27 (90%) rats completed the study and 3 rats died of complications such as malnutrition and pneumonia. In the control group, 10 (100%) rats completed the study.

### 3.2. Macroscopic Findings (Figure 2)

The middle and lower esophagus of animals in the EDA group was wide and thickened. The animals usually had very sever inflammation across the whole esophagus, manifested by esophageal shortening, enlargement of the esophageal cavity (especially the lower and middle parts), hyperkeratinization, and large-area epithelial sloughing and ulceration.

The mucosa showed longitudinal zone changes: a nodular but smooth and glistening surface giving it a cobble-stone appearance in the lower portion, nodular and uneven surface with erosion in the middle portion, and normal mucosa in the upper portion.

There was a small polypoid tumor in the lower esophagus in the EDA group. The tumor was squamous cell carcinoma and adenocarcinoma. Most of the nodular lesions were also associated with carcinomas, the others with esophagitis.

### 3.3. Microscopic Findings (Figure 3)

The esophagus of the control rats did not reveal any pathological findings but various squamous cell lesions were seen in the middle and lower esophagus in the EDA group.

As shown in Table 1, all animals from the EDA group showed histologic features of esophagitis including marked hyperplastic changes, increased thickness of the squamous epithelium, hyperkeratosis and regenerative changes with papillomatosis, and basal cell hyperplasia. These features were not found in the control group. Columnar lined epithelium (CLE) developed in distal portion of the esophagi, that is, the squamous epithelium was replaced by columnar cell lined epithelium comprising absorptive cells with brush borders. CLE was observed in 40% at the 40th week. Sever dysplasia in the lower esophagus occurred in 100%, squamous cell carcinoma was observed in 40%, and adenocarcinoma was observed in 30% at the 40th week.

To assess the biological behavior of various squamous lesions, we performed immunohistochemical staining for PCNA because the proliferative index is often increased in dysplastic and cancer tissues. PCNA labeling index of dysplasia and cancer (75 ± 5) was higher than that of control (30 ± 5).

### 3.4. Immunohistochemistry of COX2 (Figure 4)

Every animal that suffered from reflux demonstrated COX2 protein expression in the lower esophagus. COX2 immunoreactivity was mainly observed in infiltrating cells and fibroblasts in the stroma. There were some epithelial cells of SCC and ADC which strongly expressed COX2 protein.

### 3.5. Immunohistochemistry of Wild-Type p53 (Figure 5)

Wild-type p53 protein accumulation was observed as a positive nuclear staining in ADC, while it was negative in SCC.

## 4. Discussion

The present investigation demonstrates that it is duodenal contents, and not gastric contents, that induce esophageal carcinogenesis through reflux. Since this carcinogenesis required no administration of carcinogens and since spontaneous esophageal carcinoma is rare in animals, duodenal contents are probably carcinogenic in the development of esophageal carcinoma.

The histological pattern of esophageal carcinoma induced in the present study was classified into 2 types: adenocarcinoma and squamous cell carcinoma.

The adenocarcinoma always occurr near the esophagoduodenostomy and always with the columnar lined epithelium. Human esophageal adenocarcinoma mostly arises in the lower third of the esophagus, and when it does, occur it is usually associated with Barrett's esophagus. The majority of Barrett's esophagus cases result from chronic gastroesophageal reflux. SCC was observed distant from the site of anastomosis and surrounded by chronic squamous esophagitis with features of basal-cell hyperplasia and regenerative thickening.

It is widely accepted in humans that duodenal content regurgitation is closely linked to Barrett's esophagus and to the development of esophageal ADC; esophageal SCC is not reported to be related to reflux [4] but is strongly associated with tobacco smoking and alcohol consumption. Gastroesophageal reflux does not appear to be an independent risk factor for esophageal SCC but may enhance the acknowledged risk factors such as tobacco smoking and alcohol consumption. In contrast, results of several studies using rat duodenal content reflux models have shown that development of esophageal carcinomas includes squamous cell carcinoma [5]. In this study, the incidence of pure adenocarcinoma is lower than that of squamous cell carcinoma. It is unclear what factors lead to the formation of carcinomas of specified histology. Miwa et al. [6] suggested that SCC developed in places distant from the anastomosis compared to ADC. This means that histological features may depend on the volume of reflux contents; small amounts of reflux cause SCC, and a large volume of reflux causes ADC. In Mukaisho's modified model [7], they added a serosal suture between the esophagus and the jejunum after esophagojejunostomy. This addition of a serosal suture may decrease the reflux of duodenal contents compared with other models, so that the incidence of SCC was higher than that of ADC in their study. They conclude that duodenal content reflux has a great potential for malignant initiation and plays a role in developing not only ADC but also SCC.

Long-term use of NSAIDS in rheumatic patients is related to a reduced risk of various human cancers, including esophageal cancer [8]. A large body of genetic and biochemical evidence supports a role for COX2 in human and rodent tumors [9].

COX2 is well established as playing an important role in the tumorigenesis of a variety of human carcinomas and their precursor lesions. The present study demonstrated that the persistent inflammation because of duodenal reflux may promote the process from DYS to SCC or CLE to ADC. At week 40, we encountered DYS in 100%, SCC in 33%, and ADC in 35% of ED models.

In this sequence COX2 was upregulated, and cell proliferation was accelerated in the esophageal epithelium. The role of COX2 in carcinogenesis has been investigated in various carcinomas. With regard to esophageal carcinogenesis, increased COX2 expression in BE, SCC, and ADC has been reported [10]. Zimmermann et al. observed that COX2 expression was revealed immunohistochemically in 91% of 172 squamous cell carcinomas and in 78% of 27 adenocarcinomas and suggested that COX2-derived prostaglandins might play an important role in the regulation of proliferation of esophageal tumor cells [11].

In the present study, we observed a significant elevated COX2 expression in SCC and ADC. These results are in accordance with the increased COX2 expression reported for other human cancers, such as lung cancer [12], colon cancer [13], pancreatic cancer, and stomach cancer [14].

Interactions between COX2 and p53 have been shown in vitro and in vivo. It has been demonstrated that p53 can upregulate COX2 [15] or suppress the transcription of COX2 [16]. Additionally, COX2 has been observed to exhibit strong inhibitory effects on p53 transcriptional activity [17]. Benoit et al. [18] found a correlation between COX2 expression and TP53 wild-type status in esophageal adenocarcinoma with Barrett's esophagus as a precursor lesion, but not in SCC, providing evidence that the participation of p53 in the regulation of COX2 expression in cancer may be dependent on tumor histology. Esophageal cancer occurs in 2 major histopathological forms, ADC, that develops from a precursor, inflammatory metaplastic lesion, Barrett's esophagus, and SCC, that develops from the normal mucosa through a classical hyperplasia-dysplasia-carcinoma sequence. As compared with ADC, SCC is less frequently inflammatory. They proposed also, interestingly, that chronic inflammation could represent a physiopathological context in which p53 and the transcription factor NF-kappaB could cooperate to activate COX2. Our present results suggest an association between p53 accumulation and COX2 expression in ADC, with no such relation seen in SCC. On the basis of the mechanisms envisaged for the interplay between COX2 and p53 [19], it seems likely that partially different and partially shared conditions and regulatory events of COX2 and p53 expressions prevail in ADC and SCC histologies of esophageal cancer.

These results suggest that wild-type p53 participates in the upregulation of COX2 in ADC, but not in SCC.

## Figures and Tables

**Figure 2:**
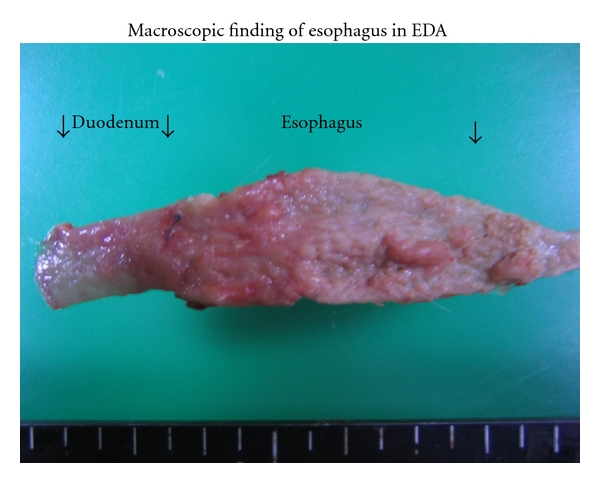
Macroscopic appearance of the esophagus in rats autopsied 40 weeks after surgery.

**Figure 3:**
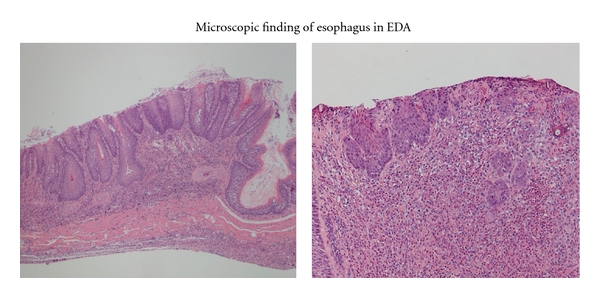
Microscopic findings in the distal portion of the esophagus in rats autopsied 40 weeks after surgery.

**Figure 4:**
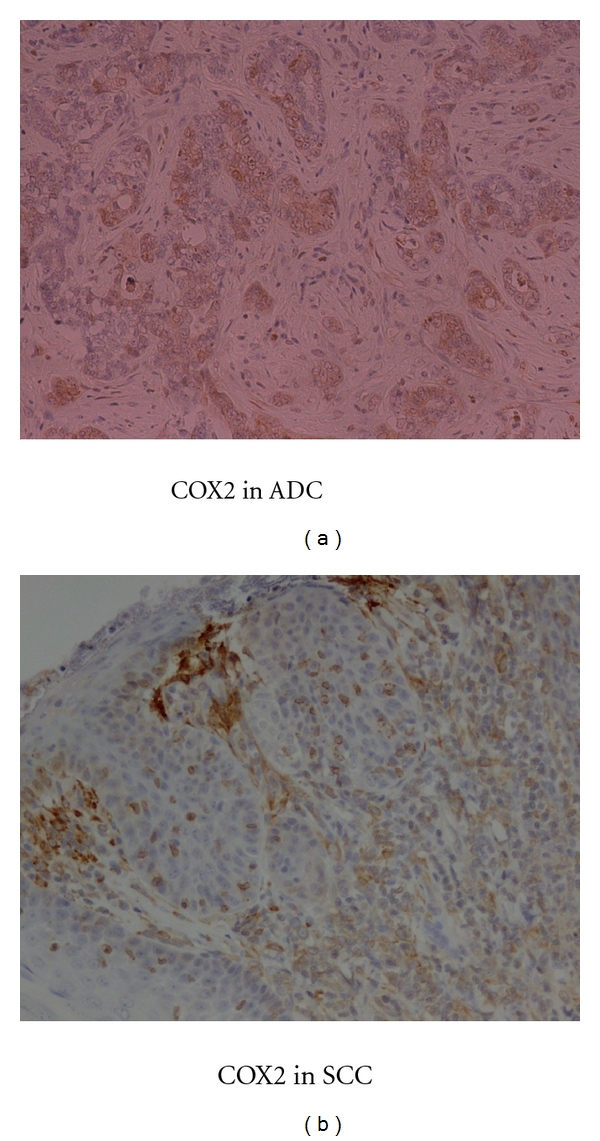
Immunohistochemical staining for COX2 in SCC and ADC.

**Figure 5:**
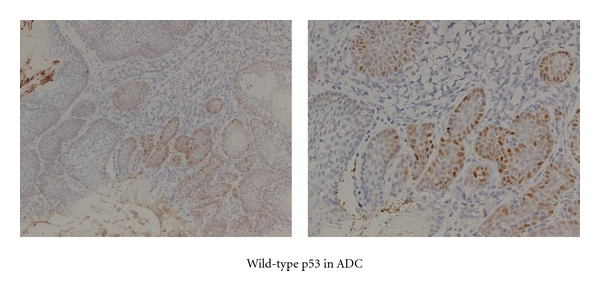
Immunohistochemical staining for wild type p53 in SCC and ADC.

**Table 1:** Incidence of histological lesions of esophagus.

Postoperative week	Group	Erosion	Regenerative hyperplasia	CLE	Dysplasia	SCC	ADC
40 w	EDA (*n* = 27)	100%	100%	40%	100%	40%	30%

EDA: esophagoduodenostomy, CLE: Columnar line epithelium, SCC: squamous cell carcinoma, ADC: Adenocarcinoma.
